# Mental Health of North Korean Refugees in Protective Facilities in China

**DOI:** 10.4306/pi.2008.5.2.70

**Published:** 2008-06-30

**Authors:** Shi-Eun Yu, Woo-Teak Jeon

**Affiliations:** 1 Korean Unification Studies, Yonsei University, Seoul, Korea.; 2 Department of Psychiatry and Medical Education, Yonsei University College of Medicine, Seoul, Korea.

**Keywords:** North Korean refugees in China, Mental health

## Abstract

**Objective:**

This study aims to provide alternative care plans for mental health of North Korean refugees who are in protective facilities in China.

**Methods:**

Personality Assessment Inventory (PAI) was utilized to measure the presence/absence of post traumatic stress disorder (PTSD) among 65 North Korean refugees.

**Results:**

The gender differences in PAI t-scores showed that women exhibited meaningfully higher scores largely in anxiety (m=61.85), depression (m=65.23), and schizophrenia (m=60.98). In different age groups, schizophrenia in the 30 age bracket (m=65.23) was meaningfully higher than the teens (m=48.11). Aggression among the treatment features was the highest in the 20 age group (m=59.19) showing higher t-scores than the teens (m=39.67). Duration in the facility affected mental health in that the 3-5 years group (m=63.91) reported the highest in paranoia. Groups of under 1 year and less than 1-3 years showed meaningfully higher scores in nonsupport. The PTSD (including partial PTSD) rate of the group recorded 9.2%. Correlation between the PTSD and PAI scores showed that the full-PTSD group demonstrated higher average scores in negative impression, somatic complaints, anxiety, anxiety-related disorder, depression, paranoia, schizophrenia, antisocial features, suicide ideation, and treatment rejection than the non-PTSD group.

**Conclusion:**

Mental health of North Korean refugees in China was worse in women, the thirties, and less than 3-5 years in the facility, and it deteriorated as the duration prolonged. To promote better psychological health of North Korean refugees in China, the attention and aid from the protection facilities and domestic and international interests are required.

## Introduction

The number of defecting North Koreans since 1990s has significantly increased due to the collapse of socialist system and North Korean economic crisis. North Korean refugees in South Korea have reached over 10,000,^1^ and those in transit in China are estimated to be over 100,000.^2^ North Korean refugees are experiencing physical and psychological human rights violations due to Chinese and North Korean governments' forced repatriation. The traumatic stress disorders they experience in the third country negatively influence the mental health of North Korean refugees even after they settle in South Korea.^3^,^4^ However, most mental health studies on North Korean refugees have focused on those who are in South Korea.^5^,^6^

Therefore, this study was conducted to find out the mental health conditions of North Korean refugees in China. The significance of this study lies in that it reflects the experiences and traumas they encounter in the process of traveling through North Korea, third country, and refugee status declaration. The target population of this study was North Korean refugees who came of the danger of being repatriated by North Korean and/or Chinese police but have not reached final place of refuge, hence in transitional. Since 2005, Chinese government strategically increased the time of encampment in protective facilities to subdue the massive escape of North Korean refugees. Therefore, North Korean refugees in protective facilities had to face longer separation from their families and serious psychological health problems. Kinzie et al. reported in their study with 13 North Korean refugees in the United States who experienced an imprisonment period in Cambodia that all refugees exhibited depression, somatic complaints, attention disorder, sleep disorder, loss of appetite, and other symptoms despite the lapse of time of trauma.^7^ Similar result was found in Carlson's study which reported that depression and post traumatic stress disorder (PTSD) were high among refugees. Carlson claimed in his study with Cambodian refugees in the U.S., that 86% of them showed PTSD symptom, and among them, 96% exhibited Harry Syndrome. Also, depression rate was above 80%.^8^ The study with Cambodian refugees in the refugee camps by Mollica also stated that 15% suffered from PTSD, 55% from depression, and 80% suffered from somatic complaints.^9^

According to studies on North Korean refugee mental health in South Korea done by Doctors without Borders with 476 North Korean refugees, 37.6% experienced psychological problems. Among these, 18.2% experienced PTSD, 18.8% complained of anxiety disorder, and 22.2% suffered from depression.^10^ Hong reported in his study that North Korean refugees who lived in South Korea for less than six months showed 27.2% full-PTSD, but after three years, the percentage declined to 4.0% which showed natural healing.^11^ Seo et al. in their 1998 study with North Korean refugees in China reported that 99% of their study group reported fear and anxiety, and around 25% said they were depressed all the time or on daily basis.^12^ Such study results show various aspects of refugees including age, gender, residential environment, adaptation during the escape, and mental health. Therefore, this research aims to establish effective mental health care system at the protective facilities by studying mental health of North Korean refugees in China, whose statuses are unstable.

## Methods

### Study group

This research initially targeted 71 North Korean refugees over 15 years of age who were in the protective facilities in China under the South Korean government protection. The purpose and contents of the study were explained to all 71 North Korean refugees prior to conducting any survey. In the end, the total number of 65 North Korean refugees agreed to participate in the study.

### Methods of the research

This research was conducted in April 21-25, 2006 and in June 1-10, 2006 in the areas of Chinese cities A and B.* The researcher visited the sites to complete interviews and survey. To avoid any loss of survey questionnaires and duplicate items, the participants were asked to complete the questionnaire and turn it in to the researchers personally and wait until the researcher reviewed the forms.

### Evaluation

The survey questions were divided largely in three parts. The first part made up of demographic details of North Korean refugees in protective facilities in China. The details included in this part were age, gender, types of residence in North Korea, time of departure from North Korea, duration of stay in China, accompaniment of family members, families in North Korea, China, and South Korea, marital status, employment, military experience, and party membership. The second part tested the mental health of North Korean refugees in China. For objective personality measurement, Morey's Personality Assessment Inventory (PAI) was utilized.^13^ Kim et al. standardized the Korean version of PAI, and the concise version with 160 questions^14^ was used for this study among the standardized Korean personality measuring inventories. This PAI included 21 scales which included validity, clinical, treatment, interpersonal scales with internal consistency of 0.76 and the median testretest reliability value of 0.79. Before conducting the research, the Korean version of PAI scales was tested with 5 North Korean refugees in South Korea. For words that North Koreans refugees reported incomprehensible, explanations were provided in parenthesis. The last section of the survey consisted of the Korean version of structural clinical interview of DSM-IV (SCID)^15^ to measure the presence of PTSD among the participants.

## Results

### Demographic characteristics and the Personality Assessment Inventory results

In gender differences, women scored higher in anxiety, depression, and schizophrenia (Table 1). More specifically, women exhibited significantly higher level of anxiety (m=61.85) than men (m=51.61). In depression also, women (m=60.72) reported meaningfully higher scores than men (m=52.32). Schizophrenia in women (m=60.89) was also higher than in men (m=52.26). These results reflect the fact that women were timid or less interested in social relationship with others in comparison with men. Women again demonstrated higher t-scores in clinical scales than men. Among them, women reported higher scores in somatic complaints (m=57.89), anxiety-related disorders (m=59.20), drug problems (m=62.41), and suicide ideation (m=53.80) than men. On the other hand, men were more anti-social (m=58.26) and rejected treatment (m=46.37) more than women did.

In terms of depression, the participants whose t-score was above 65 were 19 (29%), and among them women were 17 (89%). This result signifies that women tend to suffer more from depression, and the causes of depression originated from the separation from the family and her child(ren) in North Korea or China and subsequent fears for them.

According to the PAI validity scales among different age groups of North Korea refugees, inconsistency, infrequency, negative impression, and positive impression did not show meaningful differences. However, in clinical scales, the age differences in schizophrenia showed meaningful differences (Table 2) in that the teens reported lower t-scores (m=48.11) than the 30s age group (m=65.23). Borderline scale showed meaningful differences in all age groups. The twenties group showed higher level of anxiety-related disorder compared to other age groups and could exhibit compulsive and threatening behavior. The 30s age group scored over 60 points in depression. The reasons for high depression in the thirties might be resulted from unhappy feelings, helplessness, uncertainty of future, sleep disorder, nervousness, fears, worries, and/or emotional instability.

In the PAI treatment scales for different age groups, the t-scores for teens (m=39.67) was much lower than the twenties (m=59.19), thirties (m=56.85), and the forties (m=55.44) which showed meaningful differences in all age groups. According to the interpersonal scales among the age groups, the teens scored lowest in dominance (m=42.67) and warmth (m=39.56).

The correlation between PAI scales and duration of stay in China of North Korean refugees showed meaningful results only in paranoia and nonsupport (Table 3). In paranoia, the group that spent 3-5 years (m=63.91) showed meaningfully higher results than the group that spent under 1-3 years (m=48.06). Among the symptoms that reflected light psychopath was fear, and among disorders that indicated more serious psychopath, paranoia was observed. The nonsupport scale was reported to be high in the group that spent less than one year (m=58.57) than the group with 1-3 years. The duration of stay in China did not contribute to meaningful differences. However, interestingly the 3-5 year group's mental health in overall was worse than all the other groups. Particularly t-scores of this group in anxiety, anxiety-related disorder, schizophrenia, depression, anti-social features, and drug problems were over 60. The drug problems were reported in the groups that spent less than 1 year and over 5 years, and these participants exhibited symptoms of drug dependence such as somatic disorder, headache from stress, indigestion, and chest pain.

### Contributory factors to the Personality Assessment Inventory scales of North Korean refugees in China

The affectability of individual age, duration of stay in China, and duration under protection to the PAI scales were investigated. Negative impression scale was negatively related to duration under protection (-0.270), and warmth was positively correlated with duration of stay in China (0.268) (Table 4). In short, as the duration of stay in the protective facility prolonged, North Korean refugees frequently complained or exaggerated of physical pains. Another factor that was less meaningful was infrequency which was slightly correlated with the duration under protection (-0.236). Positive impression (0.198) increased as the duration of stay in China prolonged. This result indicates that the North Korean refugees' tendency to befriend other and avoid.

### Correlation between post traumatic stress disorder and Personality Assessment Inventory scales

According to the test for PTSD level among participating North Korean refugees in protective facilities in China, 9.2% showed symptoms of PTSD, including partial-PTSD. The result of χ^2^ validation for PTSD between genders showed no meaningful differences. The test revealed that 1 male (5.3%) and 2 female (4.3%) were diagnosed with full-PTSD, and 2 males (10.5%) and four females (8.7%) were diagnosed with partial-PTSD.

The correlations between PTSD level and PAI scales showed that the full-PTSD group showed meaningfully higher scores in negative impression (m=68.67), positive impression (m=33.67), somatic complaints (m=71.33), anxiety (m=80.33), anxiety-related disorder (m=73.33), depression (m=69.67), paranoia (m=70.00), schizophrenia (m=68.67), borderline features (m=73.67), antisocial features (m=65.00), suicide ideation (m=74.33), and treatment rejection (m=28.00) than the non-PTSD group. Particularly depression (m=80.33) was the highest, and related to somatic complaints, dependency on treatment was strong (Table 5).

Secondly, the average value of PAI scales and PTSD level, including partial-PTSD, was calculated. Compared to non-PTSD group, partial PTSD group exhibited higher level of anxiety (m=73.50), anxiety-related disorder (m=67.00), and drug problems (m=71.00). Other scales that showed differences, although not significant, were somatic complaints (m=65.50), borderline disorder (m=62.00), and suicide ideation (m=62.33).

## Discussion

The study results revealed that the North Korean refugees in protective facilities in China sowed the highest scores in drug problems (m=60.59) scale, the lowest scores in treatment rejection (m=38.38) scale, and in all scales showed under 60 points in t-scores. These results indicate the tendency to highly depend on drugs due to somatic complaints of North Korean refugees. Similar results were found in the PAI results Park et al. found in a study with prisoners under sentence.^16^ However, this result contradicts the study results from Jeon Woo-Teak's PAI study with North Korean refugees at Hanawon where depression (m=62.51) and schizophrenia (m=61.25) levels were the highest.^6^ This result could be due to their heightened emotional status after arriving at a refuge in South Korea. The PAI scores according to gender differences showed that female North Korean refugees in China experienced higher level of anxiety (m=61.85), depression (m=60.72), and schizophrenia (m=60.98) compared to males. The results from Jeon Woo Taek's PAI study with North Korean refugees at Hanawon concluded that male North Korean refugees exhibited higher level of alcohol problems, nonsupport, and warmth.^6^ Also, in an MMPI study by Kim et al. with North Korean refugees at Hanawon concluded that males scored higher in depression and schizophrenia than females.^5^ Cho stated in a depression panel study with North Korean refugees in South Korea that depression level among males significantly increased compared to females.^17^ However, Han et al. claimed in their study that the gender differentials did not result in significant discrepancy.^18^ The reasons for the change over time could be inferred that in case of females in protective facilities in China, the scores in anxiety, depression, and schizophrenia increased due to the fear from for the remaining family members in North Korea and China. On the other hand, in the case of males in Hanawon and in South Korea experience relatively high level of deprivation, unemployment, and loss of male authority compared to females.

The PAI results of North Korean refugees in this study according to age differentials showed that the teens (m=48.11) and the 30s (m=65.23) exhibited significant differences in schizophrenia. In Kim's study, the ages did not contribute to any significant differences, but the teens showed the lowest average t-scores, which was similar in this study.^5^ Roh concluded in his SCL-90-R study with North Korean refugees in Hanawon that mental health among different age groups showed lower average scores in the twenties; whereas, the forties exhibited significantly high scores in depression, anxiety, hostility, fear, and in paranoia.^19^ This conclusion is similar to the result of this study, and the increased new hopes for future plans and expectation from new life among younger generations contributed to this result. But in the 30s and 40s, limitations to employment, anxiety for unfamiliar environments in South Korea, and longing for separated families in North Korea negatively influenced their mental health.

Analysis on correlation between the PAI scales and duration of stay in China revealed that in the 3-5 year group higher level of paranoia (m=63.91) and nonsupport (m=54.18) were found. More specifically among 21 scales, the 3-5 year group exhibited the worst mental health status, even though the result was not significant. On the other hand, Chae reported in his study with North Korean refugees in South Korea that as the duration of stay in China lengthened, the tendency to prefer South Korean culture increased.^20^ Lee also claimed that North Korean refugees in South Korea with 3-5 years of experience in a foreign country reported higher satisfaction level with the life in South Korea.^21^ The 3-5 years of stay in protective facilities in China influenced North Korean refugees negatively while they were staying in the facilities. However, this period influenced positively to the adjustment to life in South Korea. In other words, the North Korean refugees who spend 3-5 years in third country in transit experience increased level of guilt for child rearing and anxiety for new life which contribute to low mental health status. However, after they arrive in Korea, in retrospect, this period turn out to give North Korean refugees an opportunity to understand South Korea and capitalism.

The PTSD level of the participants in this study was 9.4%, including partial PTSD. According to gender differentials, males (10.5%) exhibited higher level of PTSD than females (8.7%). Jeon reported in the Hanawon study that the PTSD level including partial PTSD was 8.1%. The gender differentials showed that males (2.9%) showed lower level of PTSD than females (14.3%). In comparison with this study, the overall percentage of illness was similar, but the level of PTSD among females was significantly higher in Hanawon. In addition, Hong reported in his study that the partial PTSD level among North Korean refugees with one-year-experience in South Korea was 31.8% and full PTSD was 27.2%.^11^ However, the percentage of PTSD in gender differentials was similar to this study. Kinzie et al. reported that among 322 refugees, 70% matched up with DSM-III-R PTSD standards, and among these seventy percent, 66% were females.^7^

To summarize these results, in case of North Korean refugees in China, females in gender, the thirties in age bracket, 3-5 years in duration of stay in China, and longer period in protective facilities showed poor mental health.

This study contains limitations. First, participants of this study were selected from facilities that allowed this study. Currently the overseas protective facilities are located in China, Thailand, Mongolia, and Russia. Therefore, it is hard to normalize this study to mental health report of all North Korean refugees in protective facilities. Another limitation has to do with inconsistency of administration and environment of these facilities. The protective facilities are under Chinese government surveillance, and due to special relations with North Korea, the facilities are not in a systematic structure. In addition, the administrative methods, human resources structure, and duration of protection are dissimilar, so it is hard to generalize this study. Third, the interviews and survey were conducted in rest areas and/or living areas rather than in a conference room due to infrastructural problems. It is suspected that the interviewees may not have responded honestly due to an environmental factor. Fourth, the duration of the researcher at the facilities was not sufficient to participate and observe interactions among North Korean refugees in protective facilities in China. Despite these shortcomings, the significance of the study lies in that first, this is the first North Korean refugees mental health study conducted in protective facilities in China. Second, this study offers foundational data through examining mental health status of North Korean refugees in different stages of their sojourn which can be used as the basis for mental health promotion among the refugees in South Korea. Even though this study could not cover all North Korean refugees in protective facilities in China, this study is meaningful in that it provided mental health status of North Korean refugees in different stages of refuge. Third, this research can be utilized as mental health support data in case of development of refugee camps if a large number of North Korean refugees stream into China in the future.

## Figures and Tables

**TABLE 1:**
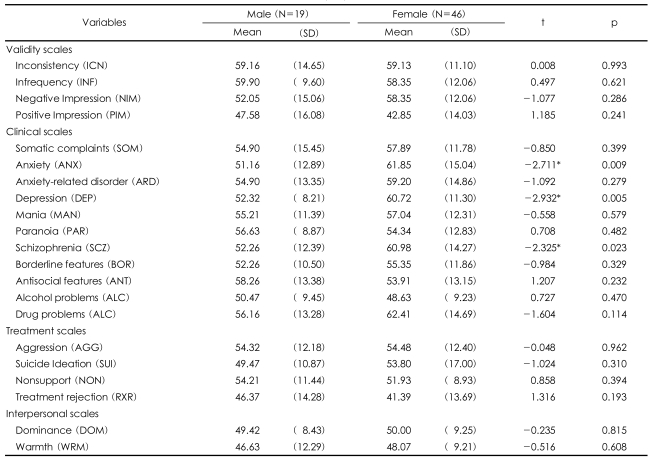
Gender differences and the Personality Assessment Index (PAI) scale

^*^p<0.05

**TABLE 2:**
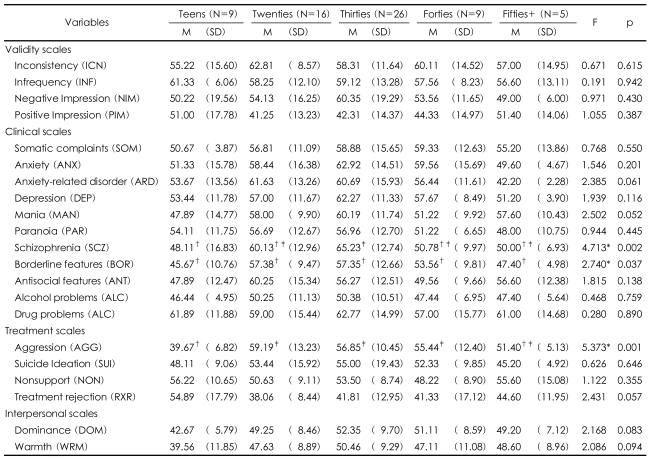
PAI scales for age groups

^*^p<0.05, ^†^^‡^Subset for alpha=0.05

**TABLE 3:**
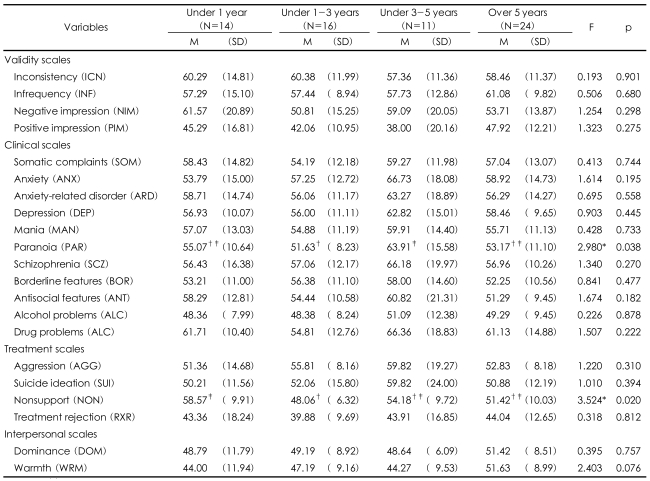
PAI scales according to duration of stay in China

^*^p<0.05, ^†^^‡^Subset for alpha=0.05

**TABLE 4:**
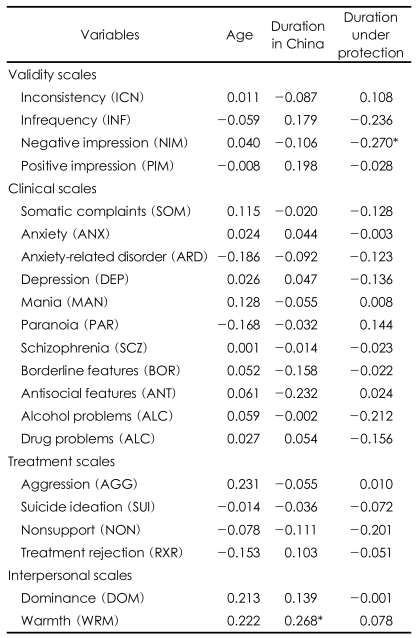
Correlations between PAI scales and age, duration in China, and duration under protection

^*^Correlation is significant at the 0.05 level (2-tailed). PAI: Personality Assessment Inventory

**TABLE 5:**
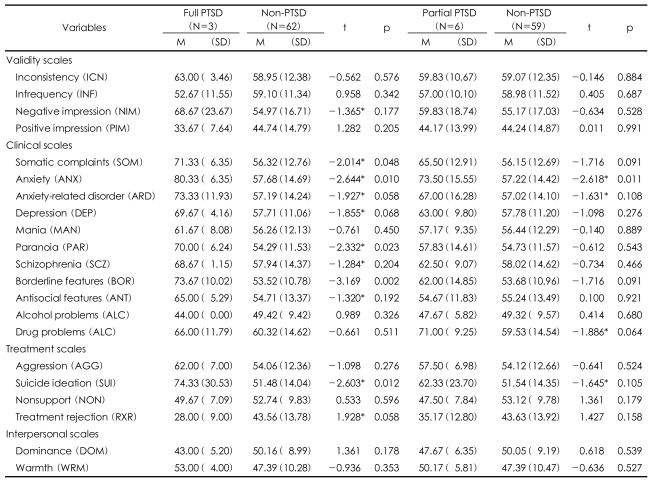
Correlation between PTSD level and PAI scales

^*^p<0.05. PTSD: post trauma stress disorder, PAI: Personality Assessment Inventory

## Footnotes

^*^The reason for using unidentifiable names for the location of the study was due to security reasons for protecting areas, facilities, size, and administrative details.
